# Family Accommodation and Preschool Dysregulated Fear Interact to Predict Child Anxiety

**DOI:** 10.1007/s10802-026-01517-z

**Published:** 2026-10-27

**Authors:** Elizabeth M. Aaron, Vivian Conran, Elizabeth J. Kiel

**Affiliations:** Department of Psychology, Miami University, 90 N. Patterson Ave, Oxford, OH 45056 United States of America

**Keywords:** Parenting, Temperament, Anxiety, Preschool, Etiology

## Abstract

Family accommodation, or behaviors that parents enact to alleviate or prevent child anxiety, has been linked to child anxiety severity. Accommodation has also been implicated in the etiology and maintenance of child anxiety. Preschool children, who may show early anxiety symptoms, may be particularly impacted by accommodation when they show temperamental predisposition towards anxiety. Dysregulated fear, characterized by displays of fear and shyness in low-threat contexts, has emerged as a temperamental construct that highlights risk for anxiety. Typically studied in toddlerhood, dysregulated fear has not been operationalized in preschool children. The current study examined whether dysregulated fear, measured observationally at child age 4, moderated the longitudinal relation from family accommodation at age 4 to change in child anxiety from age 4 to age 5. The sample included 138 children (44.5% female, 83.9% White/European American, 3.6% Hispanic/Latino) and their mothers. Dysregulated fear was coded as shyness and withdrawal during stranger approach and compliments tasks deemed “low threat” in comparison to a speech task. Mothers reported family accommodation at child age 4 and child anxiety at child ages 4 and 5. Controlling for age 4 anxiety and maternal age, there was an interaction between family accommodation and dysregulated fear in predicting child anxiety. Family accommodation related to child anxiety across values of, but most strongly at high, dysregulated fear. These results expand evidence of environment-temperament interactions in predicting child anxiety to include dysregulated fear in the preschool developmental period, which is aligned with evidence-based child anxiety prevention programs.

## Introduction

Anxiety disorders are among the most prevalent and earliest emerging mental health conditions in the general population (Liu & Perez-Edgar, 2019). It has long been recognized that preventing anxiety, rather than treating it once it develops, may set children on a trajectory of greater health and well-being as well as lower societal costs related to diagnosis and intervention (Liu & Perez-Edgar, 2019). Efficient provision of preventative intervention requires precise characterization of who is at risk. The guiding framework for the current study is the developmental psychopathology tenet that outcomes (such as anxiety symptoms) are multiply determined through various systems of influence (Rutter & Sroufe, 2000). In developmental psychopathology frameworks for anxiety (e.g., Fox et al., 2023; Liu & Perez-Edgar, 2019), multiple determination is theorized to occur through the interaction of environmental and individual factors, as in the diathesis-stress model (Monroe & Cummins, 2015). Family accommodation, which characterizes behaviors that families enact to help alleviate acute expressions of anxiety in children, has received increasing attention as a relevant environmental correlate of child anxiety in both clinical and community samples (Etkin et al., 2022; Kiel & Baumgartner, 2023; Lebowitz et al., 2013). Dysregulated fear is an individual, temperament-based trait marked by high levels of fear and distress in low-threat contexts (Buss, 2011). The construct of dysregulated fear was developed with toddlers, and existing research has focused on this developmental period. It remains unclear whether the construct continues to be relevant into the preschool period, on which many anxiety prevention programs focus (Bayer et al., 2021; Chronis-Tuscano et al., 2022). Although family accommodation and dysregulated fear each represent contemporary advances in identifying anxiety risk in their respective domains, it remains unknown whether family accommodation predicts anxiety differentially for children varying in dysregulated fear. Such knowledge is essential for theory and applied practice. Thus, the current study examined whether preschool dysregulated fear moderates the predictive association between family accommodation and child anxiety in a longitudinal study.

### Family Accommodation

Family accommodation encompasses the ways in which parents and other family members change their behavior to diminish or avoid child anxiety-related distress (Lebowitz et al., 2013). These behaviors can include parents’ active participation in the anxiety-inducing situation and modification to family routines or schedules (Etkin et al., 2022). Some common types of family accommodation include providing reassurance, speaking for the child, staying close to the child to avoid separations, and facilitating avoidance of social engagements (Etkin et al., 2022). In line with broader theory surrounding the entrenchment of behavioral patterns within families of anxiety-prone children (i.e., the anxious-coercive cycle; Dadds & Roth, 2001), family accommodation functions as negative reinforcement, resulting in the child’s continued avoidance of anxiety-producing tasks and situations over time (Etkin et al., 2022). Family accommodation is commonly observed in parents and caregivers of children with OCD and anxiety disorders (Lebowitz et al., 2013). Family accommodation has been found to be positively correlated with the severity of child anxiety symptoms (Iniesta-Sepúlveda et al., 2021), parents’ negative beliefs about anxiety (Johnco et al., 2022), and parents’ regulatory difficulties (Birk et al., 2022). Accommodation is reduced when family-involved child anxiety treatment is successful (Lebowitz et al. 2020a, ). It is important to note that most of the literature on family accommodation has been conducted in primarily White and middle-class samples, and thus generalizability of these findings to families of color and low SES families may be limited.

Although theory and empirical findings support a relation between family accommodation and child anxiety (Iniesta-Sepúlveda et al., 2021), family accommodation likely does not influence all children to the same degree. Children’s own characteristics should be considered when predicting their outcomes. Developmental models of anxiety suggests that children’s temperament may determine their susceptibility to parenting behaviors like accommodation (Fox et al., 2023; Liu & Pérez-Edgar, 2019).

### Dysregulated Fear

Temperament represents individual differences in tendencies to respond to the environment that appear early in life and have some biological basis; most temperament theories acknowledge components of both reactivity and regulation, and some include explicit focus on emotion (Gagne & Goldsmith, 2020; Pérez-Edgar, 2019). A domain of temperament historically linked to anxiety risk is behavioral inhibition (also, inhibited temperament or fearful temperament; Fox et al., 2023; Kagan et al., 1984). To be behaviorally inhibited means to be shy and withdrawn in novel and uncertain situations (Kagan et al., 1984). Behavioral inhibition is the most robust predictor of risk for anxiety later in childhood and adolescence, especially when behavioral inhibition is exhibited over time as the child ages (Liu & Perez-Edgar, 2019). However, only about one-third of behaviorally inhibited children go on to develop anxiety; inhibited/fearful temperament shows a medium effect size in predicting future anxiety (Fox et al., 2023; Sandstrom et al., 2020). This multifinality from behavioral inhibition prompted investigation into measurement refinements that could more precisely identify children at risk for anxiety.

Dysregulated fear, as a construct, developed out of this need. Dysregulated fear is the extent to which children show high levels of fear in relatively low-threat situations, reflecting difficulties in modulating the fear response to the demands of the environment (Buss, 2011; Buss et al., 2004). As originally studied, “fear” expression comprised facial fear and sadness, crying, withdrawal and lack of approach, freezing, and proximity to the mother, all of which are typical ways that toddlers express emotion and emotion regulation (Buss, 2011). The context-inappropriate nature of dysregulated fear highlights risk for maladaptive patterns of reactivity and regulation that would be expected to predict psychopathology (Buss et al., 2021). Thus, the eliciting context is crucial for the measurement of dysregulated fear, rather than the total amount of fear children show across various contexts.

Empirical evidence supports the utility of dysregulated fear in predicting anxiety outcomes among primarily White, middle-class families. Anxious behaviors in preschool and kindergarten were predicted by a dysregulated fear profile in 2-year-old toddlers above and beyond a more traditional measure of behavioral inhibition (Buss, 2011). Toddler dysregulated fear predicted peer wariness, low prosocial behavior, and observed fear in a low-threat (unfamiliar experimenter) but not high-threat (experimenter in a mask) social context in kindergarten (Buss et al., 2013). Across development, into adolescence, dysregulated fear has predicted anxiety symptomatology, even above and beyond more proximal measures of risk (Buss et al., 2021). Toddlers with a dysregulated fear profile, compared to toddlers in non-dysregulated groups, have also been shown to demonstrate higher attentional bias to threat, and toddlers with this profile who had also showed higher attentional bias had higher social withdrawal symptoms in preschool compared to toddlers with other configurations (Morales et al., 2015). Dysregulated fear seems to elicit protective parenting behaviors more so than traditional measures of temperament, and more strongly predicts social withdrawal in kindergarten through these protective parenting behaviors (Kiel & Buss, 2014).

Studies of dysregulated fear have focused on toddlerhood, but it remains unknown whether dysregulated fear can be identified in the preschool years. Preschool is the developmental period often targeted by anxiety prevention programs (e.g., Cool Little Kids, Bayer et al., 2021; the Turtle Program, Chronis-Tuscano et al., 2022). It is therefore important both to theory and to applied interventions to understand whether dysregulated fear measured in the preschool period is meaningful to the study of anxiety risk. Dysregulated fear may manifest differently in preschoolers than in toddlers. As children grow out of toddlerhood, they internalize display rules for emotion expression and may dampen facial and vocal expressions to meet perceived goals of a situation, particularly with unfamiliar people (Chaplin et al., 2017; Cole & Jacobs, 2018; Pala & Lewis, 2021). Therefore, we expected that dysregulated fear would manifest behaviorally as withdrawal, lack of approach, and low levels of interaction, which may appear more closely aligned with the construct of shyness than the discrete emotion of fear.

### Theoretical Models of Environment-Person Interaction

Contemporary developmental psychopathology theories acknowledge that environmental and individual characteristics do not act in isolation, but rather predict outcomes in interactive and dynamic ways (Humphreys et al., 2024; Sameroff, 2010). The diathesis–stress model (Zuckermann, 1999) suggests that an environmental stressor most strongly (or only) predicts a negative outcome for individuals with traits (diatheses) indicating vulnerability. In the case of the constructs of current focus, this would suggest that family accommodation would predict anxiety most strongly (or only) for children high in dysregulated fear. Theoretical models specific to children’s temperament and anxiety development (Fox et al., 2023; Liu & Pérez-Edgar, 2019) support this interactive effect while also acknowledging that temperament and parenting directly influence each other across development. Although examining reciprocal longitudinal associations between dysregulated fear and family accommodation is outside of the scope of the current study, we do not consider theoretical bidirectional relations to contradict an interactive effect in predicting a later outcome.

### Current Study

More information is needed from longitudinal studies to understand the developmental psychopathology of anxiety disorders with consideration of both family accommodation and dysregulated fear. The current study focused on preschool children to determine if dysregulated fear measured at this age is relevant to anxiety risk. We established dysregulated fear by comparing levels of shyness across tasks varying in their novelty and demands. We hypothesized an interaction effect such that family accommodation would most strongly predict anxiety one year later (controlling for earlier anxiety) for children high in dysregulated fear. Attaining knowledge about assessment of temperamental vulnerability to anxiety can modify and strengthen interventions targeting preschoolers and their families who are susceptible to the development of anxiety.

## Methods

### Participants

The sample included 138^1^ children participating in a larger longitudinal study who completed assessments at age 4 (*M* = 52.21 months, *SD* = 3.07 months) and/or 5 (*M* = 67.46, *SD* = 7.48). Of these children, 76 (55.5%) and 61 (44.5%) were female at ages 4 and 5, respectively. At the age 4 assessment, mothers ranged in age from 21.89 to 48.33 years (*M* = 34.76, *SD* = 5.34). At Phase 5, mothers ranged in age from 26.25 to 47.72 years (*M* = 36.08, *SD* = 4.85). Mothers described the child’s race as White/European American (83.9%), Black/African American (3.6%), Asian American/Pacific Islander (1.5%), multiracial (10.2%), or race not listed on the form (0.7%). Of the children, 3.6% were Hispanic/Latino. Mothers’ races were described as White/European American (93.4%), Black/African American (3.6%), Asian American/Pacific Islander (2.2%), or race not listed (0.7%). For mothers, 1.5% were Hispanic/Latino. Regarding marital status, 107 (78.1%) of the children had married parents, 10 (7.3%) of the children had divorced parents, three (2.2%) of the children had separated parents, two (1.5%) had remarried parents, and fifteen (10.9%) had single/never married mothers. Of the children in this study, 120 (87.6%) lived with both biological parents, sixteen (11.7%) lived with a single mother, and one (0.7%) lived with adopted parents. Most children (117; 85.4%) had at least one sibling. The range of years of education for mothers was from 9.00 (9th grade) to 21.00 (doctoral/professional degree; *M* = 15.82, *SD* = 2.61) years. Families ranged in income from less than $15,000 to greater than $100,000 a year, with average income being in the range of $51,000-$60,000.

### Procedure

All procedures followed ethical guidelines and received approval by the Miami University Institutional Review Board (protocol #01026r, #01245r). The study was not pre-registered (Clinical trial number: not applicable). Data and code are publicly available at https://osf.io/y75hz. (Aaron et al., (Aaron 2026)).

Children and their mothers came to the laboratory when children were approximately 4 years old. A primary experimenter explained each of the tasks and gathered written consent from the mother for herself and her child. The child then participated in the tasks while the mother completed a questionnaire battery, including surveys for family accommodation and child anxiety symptoms, in another room where she could watch her child in the tasks as they were recorded.

The child first participated in a Stranger Approach task from the Preschool Version of the Laboratory Temperament Assessment Battery (Goldsmith et al., 1999). The primary experimenter showed the child into a room and then left. After approximately 30 s, an unfamiliar research assistant entered the room and engaged the child in a conversation according to a standardized script, maintaining a neutral tone. After four questions with a 10 s pause in between each one, the stranger asked if the child had seen the primary experimenter and stated they needed papers from them. The stranger left, and after 15 s, the primary experimenter returned and asked if a person had come in. The primary experimenter stated it was their friend who was looking for papers and then left the room to look for them. The primary experimenter and stranger conversed outside of the room loudly enough for the child to overhear. The stranger then returned to the room, announced she got the papers she needed, and said goodbye in a friendly tone.

Next, children participated in a Compliments task designed to elicit shyness (Eggum-Wilkins et al., 2015). The primary experimenter led the child into the room and asked them to stand on a small platform “to see how cute you are.” The primary experimenter delivered a series of three standardized compliments, pausing in between each one and pretending to take pictures. The primary experimenter then announced that the camera was broken and it did not take any pictures.

Finally, in a Speech task adapted from procedures reported in the literature (Cobham et al., 1999; Gar & Hudson, 2008; Silk et al., 2013), the child and mother were told they had 3 min to work together to plan a speech for the child to give about their last birthday party. After 3 min of planning, the primary experimenter returned and asked if they would like to prepare a second speech about something the child learned from a book. If they did, they had another 3 min to prepare the second speech. Either immediately or after they prepared the second speech, the primary experimenter expressed excitement about hearing the speech and then instructed the child to give the speech while facing the camera. The mother was instructed to introduce herself and assist the child as necessary. The child was given 3 min to give the speech without the primary experimenter in the room. Only the first speech was coded to maintain consistency across all children in the current study.

The child and mother also engaged in additional tasks not relevant to the current study, with positive episodes and breaks occurring between the three tasks. Coders were trained by an expert coder to score the three tasks for distress and shy behavior; however, so little distress behavior occurred that the current study focuses only on the shyness scores. Coders were required to reach inter-rater reliability (intraclass correlation coefficient [ICC]) of 0.80 with the expert coder prior to coding independently. The expert coder double-scored approximately 20% of cases throughout coding to monitor and prevent coder drift. Final inter-rater reliability was determined by the ICC of the double-scoring, calculated prior to discussions about discrepant scores.

Mothers and children were invited to participate in another laboratory visit around child age 5, scheduled for the summer before the child entered kindergarten. The mother again provided written consent for her own and her child’s participation and completed a battery of questionnaires. Only questionnaire data for the anxiety outcome is used for the current study. At both the age 4 and age 5 laboratory visits, the families were compensated $50 for their time, and the child received a small thank you gift.

### Measures

#### Family Accommodation (Age 4)

The Family Accommodation Scale–Anxiety (FASA) is intended to measure the ways parents and caregivers modify their own behaviors to help diminish or reduce the child’s anxiety-relevant distress (Lebowitz et al., 2013). Mothers responded to FASA items on a Likert scale from 0 (*never*) to 4 (*daily*). The FASA has 13 total items measuring frequency of accommodations (9 items), familial distress associated with accommodation (1 item), and children’s responses to not receiving accommodation (3 items). The current study focuses on the 9 accommodation frequency items (e.g., “How often did you participate in behaviors related to your child’s anxiety?”; “Have you modified your family’s routine because of your child’s symptoms?”). Items were averaged, with higher scores indicating more accommodation. Previous research established convergent validity for the measure with strong associations between accommodation ratings and child anxiety symptoms for both parents and children; divergent validity was supported through stronger associations between family accommodation and child anxiety than child depression (Lebowitz et al. 2020a, b). The FASA showed good internal reliability in the current sample (𝛼 = .84).

#### Dysregulated Fear (Age 4)

In the Stranger Approach, Compliments, and Speech tasks, the child was scored for shyness/inhibition/withdrawal, which captured physical distancing, fidgeting, self-soothing, hiding one’s face, and/or avoiding interaction. Shyness was scored on a 1 (*none*) to 5 (*extreme shyness*,* freezing*,* and/or total avoidance*) scale. Inter-rater reliabilities were adequate for Stranger Approach (ICCs = .81 and .84 [two coders]), Compliments (ICC = .88), and Speech (ICC = .84).

Because dysregulated fear has been conceptualized as the display of fear-related behavior in contexts that do not elicit as much fear as other contexts, we compared shyness scores across the tasks to determine the episode(s) eliciting low(er) shyness than the other(s). Paired-samples *t*-tests revealed that, compared to the Speech task, children showed significantly lower shyness in the Stranger Approach (*M*_diff_ = 0.46, *SE* = 0.11, *t*[114] = 4.24, *p* < .001, Cohen’s *d* = 0.40) and Compliments (*M*_diff_ = 0.52, *SE* = 0.11, *t*[111] = 4.88, *p* < .001, Cohen’s *d* = 0.46) tasks, and the latter episodes did not differ from each other (*M*_diff_ = 0.03, *SE* = 0.11, *t*[115] = 0.31, *p* = .378, Cohen’s *d* = 0.03). Therefore, we averaged shyness scores across the Stranger Approach and Compliments tasks (*r*[114] = .30, *p* = .001) for the final variable of dysregulated fear. Shyness from the speech task was considered “high-threat” fear, relative to the lower threat episodes.

#### Child Anxiety (Ages 4 and 5)

The Preschool Anxiety Scale (PAS) is a parent-report measure of anxiety symptoms in preschoolers (Spence et al., 2001). Mothers responded to PAS items on a Likert scale from 0 (*not true at all*) to 4 (*very often true*). The PAS consists of 28 total items, with five subdimensions of Generalized Anxiety, Social Anxiety, Obsessive Compulsive Disorder, Physical Injury Fears, and Separation Anxiety. Questions included “Worries that he/she will do something to look stupid in front of other people” and “Is nervous of going swimming.” A total score can also be calculated, with higher values indicating high levels of anxiety in general. Given no hypotheses about specific aspects of anxiety, the total score (a mean of the 28 items) was used in the current study. Construct validity has previously been supported, as all scales demonstrated moderate to high correlations with the emotional symptoms scale of an established parent-report measure, indicating associations with anxiety-related symptoms (Spence et al., 2001). The PAS total score showed good internal reliability in the current sample at age 4 (𝛼 = .88) and age 5 (𝛼 = .89).

Although we used the PAS total score as a dimensional measure in primary analyses, we also translated the total scores (as the sum of items) into *t*-scores to examine representation of clinically elevated symptoms (*t*-score > 60) in this sample. Clinically-significant anxiety was endorsed for 11 children (8% of respondents) at age 4 and 18 children (13.1% of respondents) at age 5.

## Results

### Power Analyses

Given the sample size of 138, alpha of .05, and power of 0.80, G*Power version 3.1.9.7 (Faul et al., 2007) reported that a regression coefficient with an effect size as small as *f*^*2*^ = .06 (in between a small [*f*^*2*^ = .02] and medium [*f*^*2*^ = .15] effect size; Cohen, 1988) could be detected above and beyond 4 other predictors in a regression model. Previous studies report medium to large effect sizes of relations among family accommodation, dysregulated fear, and child anxiety-spectrum outcomes (Buss, 2011; Iniesta-Sepúlveda et al., 2021). Based on these estimates, the InteractionPoweR Shiny App (Baranger et al., 2023) suggested the model would be powered to detect a two-way interaction with an effect size of magnitude in between a small and medium effect (standardized beta ≈ 0.19).

### Preliminary Analyses

Descriptive statistics and bivariate correlations for primary variables are reported in Table 1. No variables exceeded thresholds for extreme deviations from a normal distribution (all skew < ❘±3❘, all kurtosis < ❘±10❘). Family accommodation related to child anxiety with roughly medium-to-large effect size both concurrently at age 4 (*r* = .43, *p* < .001) and longitudinally to age 5 (*r* = .56, *p* < .001). Dysregulated fear did not relate directly to either family accommodation or child anxiety at either age. Expectedly, child anxiety showed stability of large effect size from age 4 to age 5 (*r* = .72, *p* < .001). Anxiety scores increased from age 4 to age 5 (*t*[81] = 2.30, *p* = .024, Cohen’s *d* = 0.25).

We examined the relation of demographic variables (socioeconomic status [SES; an average of standardized scores of maternal education and household income], maternal age, child sex assigned at birth, number of siblings in the family) and age 5 child anxiety to identify potential covariates. Maternal age at the age 4 assessment significantly related to age 5 child anxiety (*r* = − .22, *p* = .033), so it was included in the primary model. All other relations were non-significant (all *p*s > .05).

Missing values occurred for dysregulated fear (*n* = 18), the age 4 surveys (*n* = 37), and age 5 child anxiety (*n* = 33), mostly because the family did not complete the assessment at that age or because they did not complete the entire battery of surveys. Missing dysregulated fear (versus not) did not relate to demographic variables or other primary variables (all *p*s > .10). Mothers missing the age 4 surveys reported significantly lower SES than mothers who completed the age 4 surveys (*M*_*diff*_ = -0.55, *SE* = 0.17, *t*[135] = -3.29, *p* < .001, Cohen’s *d* = -0.63). Families missing age 5 child anxiety had children who were reported to have lower anxiety at age 4 than families with the age 5 child anxiety variable (*M*_*diff*_ = -0.23, *SE* = 0.11, *t*[101] = -2.07, *p* = .041, Cohen’s *d* = -0.51). Age 4 child anxiety would already be included in the primary analysis, but SES was included as an auxiliary variable (a variable that assists with model estimation but is not included in the analysis itself) to reduce any missingness-related bias in the estimation of the primary model (Graham, 2009). Primary analyses handled all missing values through Full Information Maximum Likelihood estimation, so the entire sample of 138 was included.

**Table 1:** Descriptive statistics and bivariate correlations

Variable	Mean (SD)	Range	2	3	4	5	6
1. Age 4 family accommodation	0.81 (0.60)	0.00–3.89	− .14	− .09	.43***	.56***	− .21*
2. Age 4 dysregulated fear	2.13 (0.85)	1.00–5.00	--	.28**	.01	− .03	.03
3. Age 4 high-threat fear	2.60 (0.78)	1.00–4.00		--	− .10	− .17	− .13
4. Age 4 child anxiety	0.65 (0.45)	0.00–2.69			--	.72***	− .02
5. Age 5 child anxiety	0.79 (0.50)	0.00–2.97				--	− .22*
6. Maternal age	34.76 (5.34)	21.89–48.33					--

*p < .05, **p < .01, ***p < .001

### Moderation Analysis

Multiple regression analysis was run in MPlus version 7.3 (Muthén & Muthén, 1998–2015). Family accommodation and dysregulated fear were centered at their means, and their cross-product represented the interaction term. In addition to these variables, age 4 child anxiety was included as a covariate to control for existing child anxiety. As mentioned above, maternal age was also included as a covariate. Testing progressed in a top-down manner, first testing the interaction and then probing it for the change in the simple slope of family accommodation across conventional values of dysregulated fear (-1 SD, mean, + 1 SD) using recentering. Effect size of the model was estimated using Cohen’s *f*^*2*^ (small = .02, medium = .15, large = .35; Cohen, 1988). Effect sizes of individual regression coefficients were indicated indirectly by standardized regression coefficients (*ꞵ*s), values of which are estimated based on Cohen’s (1988) effect sizes for Pearson correlations (small = 0.10, medium = 0.30, large = 0.50). These standards may be too conservative when controlling for stability, which typically leads to attenuation of longitudinal predictors, which has led to the suggestion to consider even very small effect sizes (*ꞵ*s < 0.10) as meaningful (Adachi & Willoughby, 2015).

**Fig. 1:**
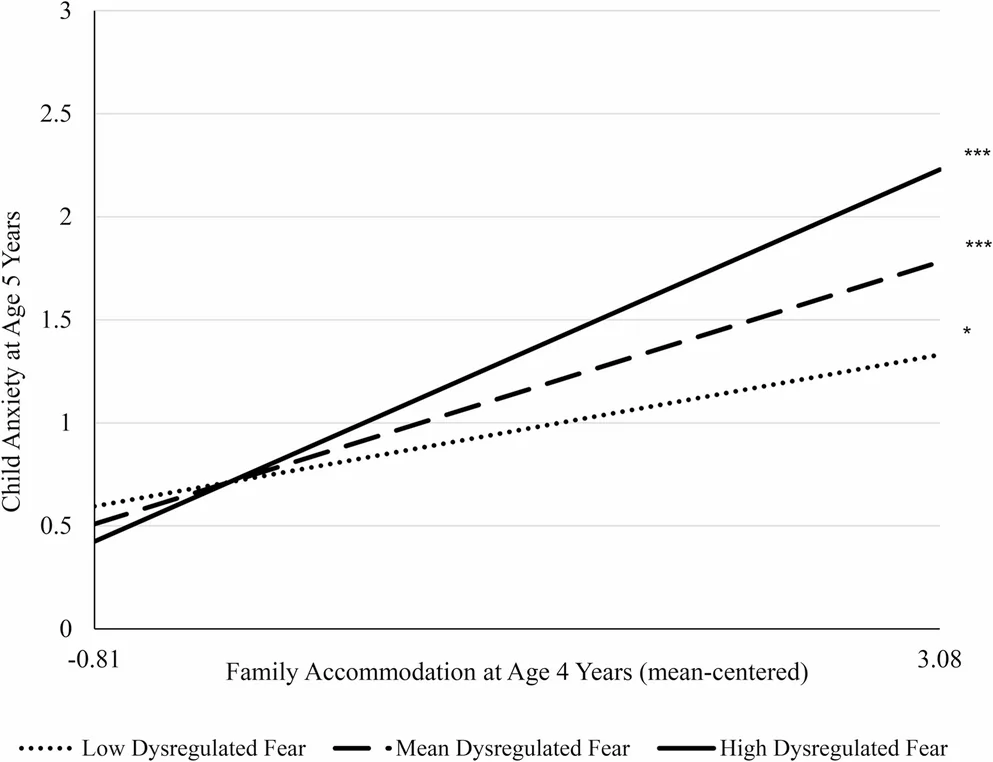
Interaction of family accommodation with dysregulated fear in predicting child anxiety. Note. Family Accommodation, dysregulated fear, and covariates (maternal age, age 4 child anxiety) were centered at their means prior to analysis. *p < .05, ***p < .001.

The overall regression model (Table 2) was significant (*R*^*2*^ = .59, *p* < .001), with proportion of variance explained exceeding standards for a large effect size (*f*^*2*^ = 1.44). The interaction between family accommodation and dysregulated fear was significant (*b* = 0.16, *SE* = 0.08, *t* = 2.07, *p* = .039) and of small effect size (*ꞵ* = 0.16). Probing the interaction (Fig. 1) revealed that the simple slope for the relation between age 4 family accommodation and age 5 child anxiety was significant across values of dysregulated fear but increased in magnitude from − 1 SD (*b* = 0.19, *SE* = 0.07, *ꞵ* = 0.23, *t* = 2.90, *p* < .001) to mean (*b* = 0.33, *SE* = 0.08, *ꞵ* = 0.40, *t* = 4.38, *p* < .001) to + 1 SD (*b* = 0.46, *SE* = 0.13, *ꞵ* = 0.57, *t* = 3.70, *p* < .001).

**Table 2:** Moderation analysis predicting age 5 child anxiety

Variable	*b* (*SE*)	*β*	*t*	*p*	95% CI
Intercept	0.77 (0.03)	--	22.77	< .001	0.71, 0.84
Maternal age	-0.01 (0.01)	-0.11	-1.47	.142	-0.02, 0.00
Age 4 child anxiety	0.57 (0.09)	0.51	6.61	< .001	0.40, 0.74
Family accommodation	0.33 (0.08)	0.40	4.38	< .001	0.18, 0.47
Dysregulated fear	0.03 (0.04)	0.05	0.79	.432	-0.05, 0.11
Family accommodation X Dysregulated fear	0.16 (0.08)	0.16	2.07	.039	0.01, 0.32

Maternal age, age 4 child anxiety, family accommodation, and dysregulated fear were centered at their means prior to analysis and prior to creation of the interaction term. The model explained a significant proportion of variance in age 5 child anxiety, *R*^*2*^ = .59, *p* < .001. Probing of the interaction is reported in text

As a sensitivity analysis, analyses were also performed with coding from the high-threat Speech task included as a covariate. In this model (*R*^*2*^ = .59, *p* < .001, *f*^*2*^ = 1.44), the interaction remained significant (*b* = 0.16, *SE* = 0.08, *t* = 2.09, *p* = .037) and high-threat fear was a non-significant predictor of child anxiety (*b* = -0.04, *SE* = 0.05, *t* = -0.85, *p* = .398).

## Discussion

Current biopsychosocial and developmental psychopathology models of anxiety support the investigation of the interaction between family environment and child temperament in relation to child anxiety development (Fox et al., 2023; Liu & Pérez-Edgar, 2019). The present study focused on family accommodation, a parenting construct that has gained attention in the child anxiety literature but whose role is less well articulated in longitudinal etiological models (Etkin et al., 2022; Kiel & Baumgartner, 2023; Lebowitz et al., 2013), and dysregulated fear, a temperament construct linked to anxiety risk (Buss, 2011; 2021) that has not yet been measured in children in developmental stages beyond toddlerhood. Overall, results were consistent with these broader theories of etiology and provide preliminary support for the relevance of these more newly developed constructs to the preschool period.

Findings supported a diathesis stress model, such that the environmental risk of family accommodation predicted child anxiety with increasing strength at increasingly higher values of dysregulated fear. Although the interaction was of relatively small effect size, this may be quite meaningful given that the analysis controlled for high stability in child anxiety (Adachi & Willoughby, 2015). Temperament, particularly the broader constructs of “difficult temperament” and negative emotionality, is supported by meta-analysis as an index of susceptibility for internalizing problems such as anxiety (Slagt et al., 2016). Research specific to anxiety etiology has shown that, in primarily White and middle-class samples, an anxiogenic caregiving environment and behavioral inhibition, a more traditional temperament construct studied for anxiety risk, interact to predict child anxiety outcomes (Fox et al., 2023; Liu & Pérez-Edgar, 2019). The mechanism of this susceptibility may be linked to attentional and learning processes, in which inhibited, compared to uninhibited, children are biased towards attending to and learning from others’ (particularly caregivers’) responses to threat (Reynolds et al., 2018). Although learning avoidant responses from family members may be most likely in early childhood (Aktar & Pérez-Edgar, 2024), our results suggest that anxiety-relevant temperament may continue to modify the impact of an anxiety-maintaining environment in preschool into the early school-age years, at least among children of White, middle-class mothers.

The significant role of dysregulated fear was maintained when controlling for wariness exhibited in a higher-threat speech task. This is consistent with foundational work with toddlers that showed a unique role of dysregulated fear in predicting future anxiety symptoms while controlling for a traditional measure of behavioral inhibition (Buss, 2011). Thus, the current findings suggest that the specificity of using low-threat contexts appears to continue to be important for preschool-age children.

However, it was not only for children high in dysregulated fear that family accommodation predicted child anxiety. This relation was present across the entire range of dysregulated fear. This is particularly salient given the age of the child anxiety assessment, which immediately preceded the transition to kindergarten for most children in the sample. The preschool and early school years are a period in which adaptive development is characterized in majority-White middle-class Western populations, which the current sample represents, by increased autonomy and engagement with others outside of the home (Denham, 2018; Dennis et al., 2007; Matte-Gangé et al., 2015). The beginning of formal schooling can be an anxiety-provoking time for both children and their parents and has been conceptualized as a period of potential risk (Hiebert-Murphy et al., 2011; Leblond et al., 2023). Children with a history of accommodation in their preschool years may not have developed the independent coping from which all children may benefit in anticipation of this transition. Of course, children at risk for anxiety begin influencing their caregiving environment early in development (Fox et al., 2023; Kiel & Baumgartner, 2023; Kiel & Buss, 2014; Liu & Pérez-Edgar, 2019); early anxiety symptoms and temperament, including dysregulated fear itself, may have been earlier drivers of the accommodation reported at the first timepoint analyzed. In any case, to facilitate independence in cultures in which it is valued, parents may engage in autonomy promoting behaviors. If these can replace accommodation, this would especially benefit children high in dysregulated fear but would likely be adaptive for all children in terms of both socioemotional and academic outcomes (Sirois et al., 2022; Vasquez et al., 2016).

Several anxiety prevention programs specifically target families of preschool-aged children. Some programs targeting children with high levels of shyness or behavioral inhibition incorporate psychoeducation about the benefits of reducing behaviors conceptually similar to accommodation (e.g., overprotection, overresponsiveness, excessive sensitivity) and increasing autonomy-supporting behavior, even including in-vivo coaching (Bayer et al., 2021; Chronis-Tuscano et al., 2015). These programs have shown reductions in anxiety symptoms and/or temperament relevant to anxiety in the range of medium to large effect sizes (Chronis-Tuscano et al., 2022). Additional parenting interventions developed specifically to reduce family accommodation (e.g., Accommodation Reduction Intervention, Kagan et al., 2023; Supportive Parenting for Anxious Childhood Emotions intervention, Lebowitz et al. 2020a, ) may be particularly beneficial for preschoolers with high dysregulated fear.

It was somewhat surprising that no direct relation was observed between preschool dysregulated fear and either family accommodation or child anxiety. When studied in toddlerhood, dysregulated fear has predicted anxiety and related problems as a main effect (Buss, 2011; Buss et al., 2013, 2021) and related concurrently to protective parenting (Kiel & Buss, 2014). It is possible that methodological differences between the dysregulated fear variable constructed in previous studies and the one used here explain these differences. Buss (2011) derived the toddler dysregulated fear variable as individual differences in the slope with which threat level of task predicted fear behaviors, with children high in dysregulated fear showing a sustained high, flat slope, and normative fear characterized by a positive slope across tasks of increasing threat. It is possible that higher-threat tasks must be incorporated in this way, statistically, to more accurately assess dysregulated fear. It is also possible that switching to solely behavioral indicators of withdrawal and lack of approach (appearing more so as shyness than discrete expressions of fear) changed the construct of dysregulated fear. However, these behaviors were included in the fear composites of toddler dysregulated fear (Buss, 2011), and the role of preschool dysregulated fear in predicting anxiety in a theoretically expected manner provides some construct validity to its measurement as such. If the variable of dysregulated fear did not capture the true range of variance of the construct in the population due to the coding scheme or the unselected nature of the sample, the effect may have been reduced and statistical power may have been attenuated. There may also have been important developmental changes between toddlerhood and preschool that we did not adequately consider. Continued longitudinal investigations of dysregulated fear from toddlerhood to preschool and beyond may assist in refining this construct in a developmentally appropriate way.

The study had limitations to be acknowledged. This study recruited a community sample which is aligned with a framework of developmental psychopathology, in which risk is identified in comparison to typical development. Although a percentage of the sample reported clinically elevated levels of anxiety at the follow-up assessment, it is unclear whether results may have been quantitatively or qualitatively different in a sample in which all children had clinical levels of anxiety and/or whose families were actively seeking treatment. For example, families of anxious individuals in clinical samples may be more likely to have family members with significant anxiety than anxious individuals from community samples (Low et al., 2008); for children, this may mean that parental and/or sibling anxiety may be more likely to be a part of the family environment than for children in our community sample. Next, the sample was mostly comprised of White, middle-class mothers and children. Parenting behaviors and their relation to anxiety differ across identity factors such as gender, race, and socioeconomic status (Cabrera & Volling, 2019; Padilla et al., 2020), and parents respond to behavioral manifestations of temperament according to culturally-informed values and socialization goals (Raval & Walker, 2019). In White, Western contexts, parents tend to value positivity, independence, and individual identity. Socialization may therefore focus on minimizing displays of anxiety and distress. Further research is therefore needed to assess the current interaction in caregivers and children with differing identities. Also potentially limiting the generalizability of the results was the moderate extent of missing values. Socioeconomic status related to missingness within the analytic sample: families lost to attrition from the larger study prior to the assessments of present focus were also lower-SES. Although bias associated with selective attrition was minimized by handling missing values according to contemporary approaches, attrition likely reduced the precision of parameter estimates, particularly for lower-SES families. Finally, the observed effect size of the tested interaction was slightly smaller than what was estimated as able to be detected from power analysis. Neither power analysis perfectly fit our regression model (G*Power [Faul et al., 2007 ] cannot account for interaction terms being more power-hungry than typical regression coefficients and InteractionPoweR [Baranger et al., 2023 ] does not account for covariates), so they may be considered approximate rather than definite. Replication of these results with a larger sample would strengthen their validity.

## Conclusion

Dysregulated fear appears to be identifiable in preschool-aged children. In line with temperament by environment interactions outlined in current theories of anxiety development, preschool dysregulated fear moderated the relation between family accommodation and change in anxiety across one year. Family accommodation predicted increased anxiety across levels of dysregulated fear but most strongly for preschoolers high in dysregulated fear. Identification of dysregulated fear in preschool-aged children may be beneficial for anxiety prevention programs targeting this developmental period.

## Data Availability

Data and code are publicly available at https://osf.io/y75hz.

## Footnotes

1 The larger longitudinal study enrolled 232 children, 4 of whom were excluded for Autism Spectrum Disorder or Neonatal Opioid Withdrawal Syndrome diagnoses. Twenty-two children were excluded because they had a sibling in the study; only 1 child per family remained in the analytic sample to preserve independence among observations. Of the remaining 206 children, 68 were lost to attrition prior to the focal assessments of this paper. These 68 families did not differ from retained families in terms of the distribution of child sex assigned at birth (*χ*2[1] = 0.04, *p* = .851) or maternal age at the time of the birth of the child (*t*[201] = 1.14, *p* = .126, Cohen’s *d* = 0.17), but, they did differ on socioeconomic status (SES; a mean of z-scored family income and maternal education): those lost to attrition had lower SES (*M* = -0.29, *SD* = 0.95) than those in the analytic sample (*M* = 0.13, *SD* = 0.87; *t*[197] = -3.06, *p* = .003, Cohen’s *d* = -0.47). Because they contributed no data to the primary analyses, these 68 children could not be included through contemporary approaches to handling missing values. SES is included as an auxiliary variable to minimize bias associated with SES-related attrition.

## References

[CR1] Aaron, E. M., Conran, V., & Kiel, E. J. (2026). *Family accommodation and preschool dysregulated fear interact to predict child anxiety*. https://osf.io/y75hz 10.1007/s10802-026-01517-z PMC13714151 42944994

[CR2] Adachi, P., & Willoughby, T. (2015). Interpreting effect sizes when controlling for stability effects in longitudinal autoregressive models: Implications for psychological science. *European Journal of Developmental Psychology*, *12*, 116–128. 10.1080/17405629.2014.963549

[CR3] Aktar, E., & Pérez-Edgar, K. (2024). Family risk factors in the acquisition of anxiety: Behavioral inhibition and social fear learning from parents. *Advances in Psychiatry and Behavioral Health*, *4*, 225–233. 10.1016/j.ypsc.2024.05.016 PMC11611299 39629334

[CR4] Baranger, D. A. A., Finsaas, M. C., Goldstein, B. L., Vize, C. E., Lynam, D. R., & Olino, T. M. (2023). Tutorial: Power analyses for interaction effects in cross-sectional regressions. *Advances in Methods and Practices in Psychological Science*, *6*(3). 10.1177/25152459231187531 PMC12341451 40799847

[CR5] Bayer, J. K., Brown, A., Prendergast, L. A., Bretherton, L., Hiscock, H., Mihalopoulos, C., Nelson-Lowe, M., Gilbertson, T., Noone, K., Bischof, N., Beechey, C., Muliadi, F., & Rapee, R. M. (2021). Follow-up of the Cool Little Kids translational trial into middle childhood. *Journal of Child Psychology and Psychiatry*, *63*, 88–98. 10.1111/jcpp.134 34128236

[CR6] Birk, S. L., Sung, J. Y., Schleider, J. L., & Olino, T. M. (2022). Unpacking parental accommodation: Relationship to parent distress tolerance and cognitive styles. *Journal of Anxiety Disorders*, *92*, 102639. 10.1016/j.janxdis.2022.102639 36274566

[CR7] Buss, K. A. (2011). Which fearful toddlers should we worry about? Context, fear regulation, and anxiety risk. *Developmental Psychology*, *47*(3), 804–819. 10.1037/a0023227 PMC3086967 21463035

[CR8] Buss, K. A., Davidson, R. J., Kalin, N. H., & Goldsmith, H. H. (2004). Context-specific freezing and associated physiological reactivity as a dysregulated fear response. *Developmental Psychology*, *40*(4), 583–594. 10.1037/0012-1649.40.4.583 15238045

[CR10] Buss, K. A., Davis, E. L., Kiel, E. J., Brooker, R. J., Beekman, C., & Early, M. C. (2013). Dysregulated fear predicts social wariness and social anxiety symptoms during kindergarten. *Journal of Clinical Child & Adolescent Psychology*, *42*(5), 603–616. 10.1080/15374416.2013.769170 PMC3675171 23458273

[CR9] Buss, K. A., Cho, S., Morales, S., McDoniel, M., Webb, A. F., Schwartz, A., Cole, P. M., Dorn, L. D., Gest, S., & Teti, D. M. (2021). Toddler dysregulated fear predicts continued risk for social anxiety symptoms in early adolescence. *Development and Psychopathology*, *33*(1), 252–263. 10.1017/S0954579419001743 PMC7483171 32115004

[CR11] Cabrera, N. J., & Volling, B. L. (2019). VIII. Moving research on fathering and children’s development forward: Priorities and recommendations for the future. *Advancing research and measurement on fathering and children’s development*, *84*, 7–17. 10.1111/mono.12404

[CR13] Chaplin, T. M., Klein, M. R., Cole, P. M., & Turpyn, C. C. (2017). Developmental change in emotion expression in frustrating situations: The roles of context and gender. *Infant and child development*, *26*(6), e2028. 10.1002/icd.2028 PMC6196744 30364529

[CR14] Chronis-Tuscano, A., , Novick, D. R., Danko, C. M., Smith, K. A., Wagner, N. J., Wang, C. H., Druskin, L., Dougherty, L. R., & Rubin, K. H. (2022). Early intervention for inhibited young children: A randomized controlled trial comparing the Turtle Program and Cool Little Kids.. *Journal of Child Psychology *& *Psychiatry*, *6**3*(3), 273-281 . 10.1111/jcpp.13475 PMC11270476 34184792

[CR15] Cobham, V. E., Dadds, M. R., & Spence, S. H. (1999). Anxious children and their parents: What do they expect? *Journal of Clinical Child Psychology*, *28*(2), 220–231. 10.1207/s15374424jccp2802_9 10353081

[CR16] Cohen, J. (1988). *Statistical power analysis for the behavioral sciences* (2nd ed.). Lawrence Erlbaum Associates,.

[CR17] Cole, P. M., & Jacobs, A. E. (2018). From children’s expressive control to emotion regulation: Looking back, looking ahead. *European Journal of Developmental Psychology*, *15*(6), 658–677. 10.1080/17405629.2018.1438888 PMC6424503 30899314

[CR20] Dadds, M. R., & Roth, J. H. (2001). Family processes in the development of anxiety problems. In M. W. Vasey, & M. R. Dadds (Eds.), *The developmental psychopathology of anxiety* (pp. 278–303). Oxford University Press.

[CR18] Denham, S. A. (2018). Implications of Carolyn Saarni’s work for preschoolers’ emotional competence. *European Journal of Developmental Psychology*, *15*(6), 643–657. 10.1080/17405629.2018.1479250

[CR19] Dennis, T. A., Talih, M., Cole, P. M., Zahn-Waxler, C., & Mizuta, I. (2007). The socialization of autonomy and relatedness: sequential verbal exchanges in Japanese and US mother—preschooler dyads. *Journal of Cross-Cultural Psychology*, *38*(6), 729–749. 10.1177/0022022107308993

[CR21] Eggum-Wilkens, N. D., Lemery-Chalfant, K., Aksan, N., & Goldsmith, H. H. (2015). Self-conscious Shyness: Growth during Toddlerhood, Strong Role of Genetics, and No Prediction from Fearful Shyness. *Infancy*, *20*, 160–188. 10.1111/infa.12070 PMC4322942 25685095

[CR22] Etkin, R. G., Silverman, W. K., & Lebowitz, E. R. (2022). Anxiety and social functioning: The moderating roles of family accommodation and youth characteristics. *Research on Child and Adolescent Psychopathology*, *50*, 781–794. 10.1007/s10802-021-00884-z 34997402

[CR23] Faul, F., Erdfelder, E., Lang, A. G., & Buchner, A. (2007). G*Power 3: A flexible statistical power analysis program for the social, behavioral, and biomedical sciences. *Behavior Research Methods*, *39*(2), 175–191. 10.3758/BF03193146 17695343

[CR24] Fox, N. A., Zeytinoglu, S., Valadez, E. A., Buzzell, G. A., Morales, S., & Henderson, H. A. (2023). Annual research review: Developmental pathways linking early behavioral inhibition to later anxiety. *The Journal of Child Psychology and Psychiatry*, *64*(4), 537–561. 10.1111/jcpp.13702 PMC10690832 36123776

[CR25] Gagne, J. R., & Goldsmith, H. H. (2020). Development of temperament in infancy and childhood. In K. J. Saudino, & J. M. Ganiban (Eds.), *Behavior genetics of temperament and personality. Advances in behavior genetics* (pp. 3–40). Springer. 10.1007/978-1-0716-0933-0_1

[CR26] Gar, N. S., & Hudson, J. L. (2008). An examination of the interactions between mothers and children with anxiety disorders. *Behaviour Research and Therapy*, *46*(12), 1266–1274. 10.1016/j.brat.2008.08.006 18851844

[CR28] Goldsmith, H. H., Reilly, J., Lemery, K. S., Longley, S., & Prescott, A. (1999). *The Laboratory Temperament Assessment Battery – Preschool Version*. University of Wisconsin-Madison.

[CR29] Graham, J. W. (2009). Missing data analysis: Making it work in the real world. *Annual Review of Psychology*, *60*(1), 549–576. 10.1146/annurev.psych.58.110405.085530 18652544

[CR30] Hiebert-Murphy, D., Williams, E. A., Mills, R. S. L., Walker, J. R., Feldgaier, S., Warren, M., Freeman, W., McIntyre, M., & Cox, B. J. (2011). Listening to parents: The challenges of parenting kindergarten-aged children who are anxious. *Clinical Child Psychology & Psychiatry*, *17*, 384–399. 10.1177/1359104511415495 21852319

[CR31] Humphreys, K. L., Garon-Bissonnette, J., Hill, K. E., Bailes, L. G., Barnett, W., & Hare, M. M. (2024). Caregiving relationships are a cornerstone of developmental psychopathology. *Development and Psychopathology*, *36*(5), 2218–2231. 10.1017/S0954579424000300 PMC11341779 38389283

[CR32] Iniesta-Sepúlveda, M., Rodriguez-Jimenez, T., Lebowitz, E. R., Goodman, W. K., & Storch, E. A. (2021). The relationship of family accommodation with pediatric anxiety severity: Meta-analytic findings and child, family and methodological moderators. *Child Psychiatry & Human Development*, *52*(1), 1–14. 10.1007/s10578-020-00987-6 32246361

[CR33] Johnco, C., Storch, E. A., Oar, E., McBride, N. M., Schneider, S., Silverman, W. K., & Lebowitz, E. R. (2022). The role of parental beliefs about anxiety and attachment on parental accommodation of child anxiety. *Research on Child and Adolescent Psychopathology*, *50*, 51–62. 10.1007/s10802-020-00722-8 33534096

[CR35] Kagan, J., Reznick, J. S., Clarke, C., Snidman, N., & Garcia–Coll, C. (1984). Behavioral inhibition to the unfamiliar. *Child Development*, *55*(6), 2212–2225. 10.2307/1129793

[CR34] Kagan, E. R., Frank, H. E., Palitz, S. A., & Kendall, P. C. (2023). Targeting parental accommodation in anxiety: An open trial of the coping cat accommodation reduction intervention. *Journal of Child and Family Studies*, *32*(2), 398–408. 10.1007/s10826-022-02419-6

[CR36] Kiel, E. J., & Baumgartner, N. M. (2023). Construct validation for toddler-solicited maternal comforting behavior as relevant to family accommodation and child anxiety risk. *Research on Child and Adolescent Psychopathology*, *51*(3), 413–425. 10.1007/s10802-022-00990-6 PMC9660142 36370221

[CR37] Kiel, E. J., & Buss, K. A. (2014). Dysregulated fear in toddlerhood predicts kindergarten social withdrawal through protective parenting. *Infant and Child Development*, *23*, 304–313. 10.1002/icd.1855 PMC4167770 25242893

[CR38] Leblond, M., Parent, S., Castellanos-Ryan, N., Lupien, S., Fraser, W. D., & Seguin, J. R. (2023). Stress across the transition from preschool to school: association with the development of anxiety symptoms in children. *Psychoneuroendocrinology*, *153*, 106144. 10.1016/j.psyneuen.2023.106144

[CR41] Lebowitz, E. R., Woolston, J., Bar-Haim, Y., Calvocoressi, L., Dauser, C., Warnick, E., Scahill, L., Chakir, A. R., Shechner, T., Hermes, H., Vitulano, L. A., King, R. A., & Leckman, J. F. (2013). Family accommodation in pediatric anxiety disorders. *Depression and Anxiety*, *30*, 47–54. 10.1002/da.21998 PMC3932435 22965863

[CR39] Lebowitz, E. R., Marin, C. E., Martino, A., Shimshoni, Y., & Silverman, W. K. (2020a). Parent-based treatment as efficacious as cognitive-behavioral therapy for childhood anxiety: A randomized noninferiority study of supportive parenting for anxious childhood emotions. *Journal of the American Academy of Child and Adolescent Psychiatry*, *59*, 362–372. 10.1016/j.jaac.2019.02.014 PMC6732048 30851397

[CR40] Lebowitz, E. R., Marin, C. E., & Silverman, W. K. (2020b). Measuring family accommodation of childhood anxiety: Confirmatory factor analysis, validity, and reliability of the parent and child family accommodation scale–anxiety. *Journal of Clinical Child & Adolescent Psychology*, *49*(6), 752–760. 10.1080/15374416.2019.1614002 PMC6881529 31136200

[CR42] Liu, P., & Pérez–Edgar, K. E. (2019). Developmental pathways from early behavioral inhibition to later anxiety: An integrative review of developmental psychopathology research and translational implications. *Adolescent Research Review*, *4*(1), 45–58. 10.1007/s40894-018-0092-5

[CR43] Low, N. C., Cui, L., & Merikangas, K. R. (2008). Community versus clinic sampling: effect on the familial aggregation of anxiety disorders. *Biological Psychiatry*, *63*(9), 884–890. 10.1016/j.biopsych.2007.08.011 17945198

[CR44] Matte-Gagné, C., Harvey, B., Stack, D. M., & Serbin, L. (2015). Contextual specificity in the relationship between maternal autonomy support and children’s socio-emotional development: A longitudinal study from preschool to preadolescence. *Journal of Youth and Adolescence*, *44*, 1528–1541. 10.1007/s10964-014-0247-z 25559414

[CR45] Monroe, S. M., & Cummins, L. F. (2015). Diathesis-stress models. In R.L. Cautin & S.O. Lilienfeld (Eds.), *The Encyclopedia of Clinical Psychology*. 10.1002/9781118625392.wbecp466

[CR46] Morales, S., Pérez-Edgar, K. E., & Buss, K. A. (2015). Attention biases towards and away from threat mark the relation between early dysregulated fear and the later emergence of social withdrawal. *Journal of Abnormal Child Psychology*, *43*(6), 1067–1078. 10.1007/s10802-014-9963-9 PMC4469642 25510354

[CR47] Muthén, L. K., & Muthén, B. O. (1998–2015). *Mplus user’s guide (Seventh Edition)*. Muthén & Muthén.

[CR48] Padilla, C. M., Hines, C. T., & Ryan, R. M. (2020). Infant temperament, parenting and behavior problems: Variation by parental education and income. *Journal of Applied Developmental Psychology*, *70*, 101179. 10.1016/j.appdev.2020.101179

[CR49] Pala, F. C., & Lewis, C. (2021). Do preschoolers grasp the importance of regulating emotional expression? *European Journal of Developmental Psychology*, *18*(4), 494–519. 10.1080/17405629.2020.1814251

[CR50] Pérez-Edgar, K. (2019). Through the looking glass: Temperament and emotion as separate and interwoven constructs. In V. LoBue, K. Pérez-Edgar, & K. A. Buss (Eds.), *Handbook of emotional development* (pp. 139–168). Springer.

[CR52] Raval, V. V., & Walker, B. L. (2019). Unpacking ‘culture’: Caregiver socialization of emotion and child functioning in diverse families. *Developmental Review*, *51*, 146–174. 10.1016/j.dr.2018.11.001

[CR53] Reynolds, G., Askew, C., & Field, A. P. (2018). Behavioral inhibition and the associative learning of fear. In: K. Pérez-Edgar & N. Fox, N. (Eds.), *Behavioral inhibition*. Cham, Switzerland: Springer. 10.1007/978-3

[CR64] Rutter, M., & Sroufe, L. A. (2000). Developmental psychopathology: Concepts and challenges. *Development and Psychopathology*, 12(3), 265–296. 10.1017/S0954579400003023 11014739

[CR59] Sameroff, A. (2010). A unified theory of development: A dialectic integration of nature and nurture. *Child development*, *81*(1), 6–22. 10.1111/j.1467-8624.2009.01378.x 20331651

[CR54] Sandstrom, A., Uher, R., & Pavlova, B. (2020). Prospective association between childhood behavioral inhibition and anxiety: A meta-analysis. *Research on Child and Adolescent Psychopathology*, *48*(1), 57–66. 10.1007/s10802-019-00588-5 31642030

[CR55] Silk, J. S., Sheeber, L., Tan, P. Z., Ladouceur, C. D., Forbes, E. E., McMakin, D. L., Dahl, R. E., Siegle, G. J., Kendall, P. C., Mannarino, A., & Ryan, N. D. (2013). You can do it! The role of parental encouragement of bravery in child anxiety treatment. *Journal of Anxiety Disorders*, *27*(5), 439–446. 10.1016/j.janxdis.2013.06.002 PMC3766422 23851000

[CR56] Sirois, M. S., Bernier, A., Gagné, C. M., & Mageau, G. A. (2022). Early maternal autonomy support as a predictor of child internalizing and externalizing behavior trajectories across early childhood. *Social Development*, *31*(3), 883–899. 10.1111/sode.12575

[CR57] Slagt, M., Dubas, J. S., Deković, M., & van Aken, M. A. (2016). Differences in sensitivity to parenting depending on child temperament: A meta-analysis. *Psychological Bulletin*, *142*(10), 1068. 10.1037/bul0000061 27513919

[CR58] Spence, S. H., Rapee, R., McDonald, C., & Ingram, M. (2001). The structure of anxiety symptoms among preschoolers. *Behaviour Research and Therapy*, *39*, 1293–1316. 10.1016/S0005-7967(00)00098-X 11686265

[CR62] Vasquez, A. C., Patall, E. A., Fong, C. J., Corrigan, A. S., & Pine, L. (2016). Parent autonomy support, academic achievement, and psychosocial functioning: A meta-analysis of research. *Educational Psychology Review*, *28*(3), 605–644. 10.1007/s10648-015-9329-z

[CR63] Zuckermann, M. (1999). *Vulnerability to psychopathology: a biosocial model*. American Psychological Association.

